# Identification of Post-myocardial Infarction Blood Expression Signatures Using Multiple Feature Selection Strategies

**DOI:** 10.3389/fphys.2020.00483

**Published:** 2020-06-03

**Authors:** Ming Li, Fuli Chen, Yaling Zhang, Yan Xiong, Qiyong Li, Hui Huang

**Affiliations:** ^1^Department of Cardiology, Eastern Hospital, Sichuan Academy of Medical Sciences & Sichuan Provincial People’s Hospital, Chengdu, China; ^2^Department of Cardiology, Sichuan Academy of Medical Sciences & Sichuan Provincial People’s Hospital, Chengdu, China; ^3^Department of Nephrology, Eastern Hospital, Sichuan Academy of Medical Sciences & Sichuan Provincial People’s Hospital, Chengdu, China

**Keywords:** myocardial infarction, Monte Carlo feature selection, incremental feature selection, support vector machine, gene

## Abstract

Myocardial infarction (MI) is a type of serious heart attack in which the blood flow to the heart is suddenly interrupted, resulting in injury to the heart muscles due to a lack of oxygen supply. Although clinical diagnosis methods can be used to identify the occurrence of MI, using the changes of molecular markers or characteristic molecules in blood to characterize the early phase and later trend of MI will help us choose a more reasonable treatment plan. Previously, comparative transcriptome studies focused on finding differentially expressed genes between MI patients and healthy people. However, signature molecules altered in different phases of MI have not been well excavated. We developed a set of computational approaches integrating multiple machine learning algorithms, including Monte Carlo feature selection (MCFS), incremental feature selection (IFS), and support vector machine (SVM), to identify gene expression characteristics on different phases of MI. 134 genes were determined to serve as features for building optimal SVM classifiers to distinguish acute MI and post-MI. Subsequently, functional enrichment analyses followed by protein-protein interaction analysis on 134 genes identified several hub genes (IL1R1, TLR2, and TLR4) associated with progression of MI, which can be used as new diagnostic molecules for MI.

## Introduction

Myocardial infarction (MI), one of the most common cardiac diseases, has been a serious threat to human health worldwide for a long period. According to the third universal definition of MI, it is the condition of myocardial necrosis in a clinical setting consistent with myocardial ischemia (Bax et al., 2012). MI occurs when the blood flow is impaired and the cardiomyocyte is injured due to the lack of oxygen supply (Lu et al., 2015). Patients with coronary atherosclerosis have a high risk of developing a MI when inflammation takes place in the vascular wall (Thygesen et al., 2007). Usually a more serious event is termed as acute myocardial infarction (AMI). The symptoms of MI include chest pain, shortness of breath, abnormal heart beating, and fatigue (Kosuge et al., 2006). Smoking and dyslipidemia are thought to be important risk factors for MI, which is correlated with the increasing mortality rate in China (Critchley et al., 2004). Approximately three million cases of MI are diagnosed every year and the annual incidence rate is about 600 cases per 100,000 people (Rogers et al., 2008; Nascimento et al., 2019). The average mortality of MI is approximately 27% according to statistics (White and Chew, 2008), making it a major cause of death in the world.

After the onset of MI, many pathological processes occur, such as the death of myocardial cells, and will develop into different conditions depending on the status of the patient. MI can be classified pathologically as acute, healing, or healed, which is roughly correlated with the disease duration. Acute MI describes a severe event usually accompanied by activated inflammation at early onset. Then it progresses to healing, which can be characterized by the presence of mononuclear cells and fibroblasts and the absence of polymorphonuclear leukocytes. The entire process reaching the healed state of MI takes about several months when cellular infiltration fades away and scar tissue appears (Thygesen et al., 2007). The different phases after onset reflect distinct pathological conditions. So, a better understanding of the phases will contribute to the treatment of MI and improve the outcomes of patients.

Early and rapid diagnosis is important for the decision of treatment and improvement of survival. There are several methods for the evaluation of MI including electrocardiography (ECG) and cardiac markers. The ECG has a high specificity of 90% for MI but a poor sensitivity of 20% (Zimetbaum and Josephson, 2003). Serum biomarkers of myocardial necrosis, such as cardiac troponin (I or T), which can specifically reflect myocardial injury, show high clinical sensitivity and can improve the diagnostic accuracy (Jaffe et al., 2000). Levels of MB isoforms of creatine (CK-MB) also exhibit the ability to identify MI as an increased CK-MB value is associated with myocarditis and electrical cardioversion (Members et al., 2007). Although the traditional clinical approach has shown excellent performance for diagnosing MI, an increasing number of studies have proven that molecular markers, like the transcription profile in serum, are capable of reflecting detailed pathological conditions and subsequent progress of MI, which will help to determine the optimal treatment.

Owing to the great development in RNA-seq technology, many novel genes are found to play crucial roles in various diseases. It has been reported that the specific expression pattern of certain genes is relevant to the pathological condition of MI. For examples, H-FABP, which is involved in myocardial fatty-acid metabolism, is rapidly released into the cytosol in early MI and can act as an early marker (Glatz et al., 1988). B-type Natriuretic Peptide (BNP) is secreted by the ventricles in response to the tension of cardiomyocytes and leads to the reduction of blood pressure, making it a prognostic marker after MI (De Lemos et al., 2001). Growth Differentiation Factor-15 (*GDF15*) is specifically expressed in the heart when ischemia or reperfusion happened, and increasing *GDF15* indicates a higher risk of death in MI patients (Wollert et al., 2007). Besides, non-coding RNAs are also found to be involved in the pathogenesis of MI. Circulating miR-208a, which is only detected in AMI patients, is thought to be the novel potential biomarker for early diagnosis with higher sensitivity and specificity (Wang et al., 2010). Given that the progress of MI involves numerous complex biological processes and pathways, the overall transcriptome analysis will contribute to revealing a more detailed molecular mechanism and an easier way to locate the key genes related to pathogenesis of MI.

In this study, we utilized bioinformatics methods to explore the key gene networks associated with MI from the vast transcriptomic data. Previous studies which aimed to find the biomarker for MI put the focus on separated genes but ignored the linkage among them. With the application of bioinformatics, we can study the complex expression network consisting of multiple genes with less time consumed and a higher efficiency. Transcriptomic data was obtained from the published paper which performed whole blood RNA profiling at different time points in cohort with MI (Vanhaverbeke et al., 2019). In order to identify the key biomarkers for distinguishing different pathological extents, we manually divided all patients into three categories based on the duration of MI. These three different groups roughly reflect distinct pathological conditions. Next, we constructed an optimal support vector machine (SVM) model with the application of a feature selection method called Monte Carlo Feature Selection (MCFS) (Chen et al., 2018a, 2019a,b, 2019d, 2020; Pan et al., 2018, 2019a,b; Wang et al., 2018; Jiang et al., 2019; Li et al., 2019) and incremental feature selection (IFS) (Chen et al., 2018b,d; Lei et al., 2018; Li and Huang, 2018; Sieber et al., 2018; Zhang et al., 2018; Wang and Huang, 2019; Yan et al., 2019). 134 optimal genes were selected which show specific expression patterns during varied phases of MI and can distinguish different categories with a highly accuracy. The functional enrichment analysis suggested the important biological processes and pathways related to the progress of MI and corresponding hub genes were identified by gene network analysis. The selected genes in the current study can serve as novel biomarkers for different phases of MI and contribute to revealing the pathological mechanism of MI.

## Materials and Methods

### Dataset

The blood gene expression profiles of 166 samples which incorporate three phases of MI (D0: acute MI, D30: 30-days post-MI, and Y1: 1-year post-MI) were downloaded with the gene expression omnibus (GEO) under accession number of GSE123342 (Vanhaverbeke et al., 2019). There were 65 D0, 64 D30, and 37 Y1 samples. There were 70,523 probes in Affymetrix Human Transcriptome Array 2.0 corresponding to 30,905 genes. The probes for the same gene were averaged and the data was quantile normalized (Bolstad et al., 2003). We wanted to find the genes with changed expression patterns in post-MI.

### Monte Carlo Feature Selection (MCFS)

Monte Carlo feature selection has been a widely used method for feature selection (Chen et al., 2018a, 2019a,b,d, 2020; Pan et al., 2018, 2019a,b; Wang et al., 2018; Jiang et al., 2019; Li et al., 2019). It was originally developed by Draminski et al. (2008). It randomly constructed many tree classifiers of the sub datasets from the original dataset and assigned the importance to a feature based on how much it participated in the tree classifiers. The java software dmLab^1^ with default parameters (Draminski et al., 2008) was used to apply the Monte-Carlo feature selection method.

To be more specific, the original dataset was divided into s subsets of m features (m<<d, where d is the total number of features, i.e., 30,905 genes in this study). Then, for each subset, t trees were constructed. Therefore, a total of s⋅t classification trees were constructed. At last, the relative importance (RI) of each feature was estimated as follows:

(1) $$R\,I_{g}=\sum_{\tau =1}^{s\,t}{\left(w\,A\,c\,c\right)}^{u}\,\sum_{n_{g}\,\left(\tau \right)}I\,G\,\left(n_{g}\,\left(\tau \right)\right)\,{\left(\frac{n\,o.i\,n\,n_{g}\,\left(\tau \right)}{n\,o.i\,n\,\tau }\right)}^{v}$$

where *IG*(*n*_*g*_(τ)) was the information gain (IG) of node *n*_*g*_(τ), (*no*.*inn*_*g*_(τ)) was the number of samples in node *n*_*g*_(τ), (*no*.*in*τ) was the number of samples in tree τ, *wAcc* was the weighted accuracy over all samples, and *u* and *v* were two regular factors which were set as default.

After running MCFS, all features can be ranked based on their RI. The higher the RI, the more important a feature was.

### Incremental Feature Selection (IFS)

With MCFS, all features were ranked. But we still did not know how many genes we should choose. Ideally, we wanted the number of selected genes to be small but their classification performance to be great. To find the balance and the optimal signature, we adopted IFS (Chen et al., 2018b,d; Lei et al., 2018; Li and Huang, 2018; Sieber et al., 2018; Zhang et al., 2018; Wang and Huang, 2019; Yan et al., 2019). During IFS, a serial of feature sets *F* = [*f*_1_,*f*_2_,…,*f*_*N*_] were constructed. N ranged from 1 to 1000. For each feature set, we constructed corresponding support vector machine (SVM) classifiers using the R function svm with default parameters in package e1071^2^ and evaluated the performance using leave-one-out cross validation (LOOCV). Therefore, we can get a serial of LOOCV accuracies which corresponded to different feature sets with various numbers of features. With the help of the IFS curve, we can balance the model complexity and classification performance. If the number of features was too small, the performance would be bad. If the number of features was too large, too much noise would be introduced and the performance would decrease. The optimal selection would be achieved when the number of features was small and the accuracy was high.

### Functional Enrichment Analysis

The biological functions of the optimal MI signature genes were analyzed using hypergeometric enrichment analysis (Shi et al., 2018a, b). The significance of the signature genes onto Kyoto Encyclopedia of Genes and Genomes (KEGG) pathways, Gene Ontology (GO) biological process (BP), molecular function (MF), and cell component (CC) were represented with hypergeometric *p* values.

## Results

### Feature Ranking Based on MCFS Method

In this study, we exploited newly published gene expression profiles of patients with MI (Vanhaverbeke et al., 2019). Each patient was represented by 30,905 gene expression features. We integrated expression profiles of all patients into one matrix for quantile normalization followed by applying the MCFS method for ranking analysis. Each feature was assessed by estimating the relative importance (RI) value. After evaluating all features, we generated a feature list *F* in descending order of RI values of features. The ranked features with RI values were provided in Supplementary Table S1.

### Establishing Classifier Using SVM With IFS

**FIGURE 1:**
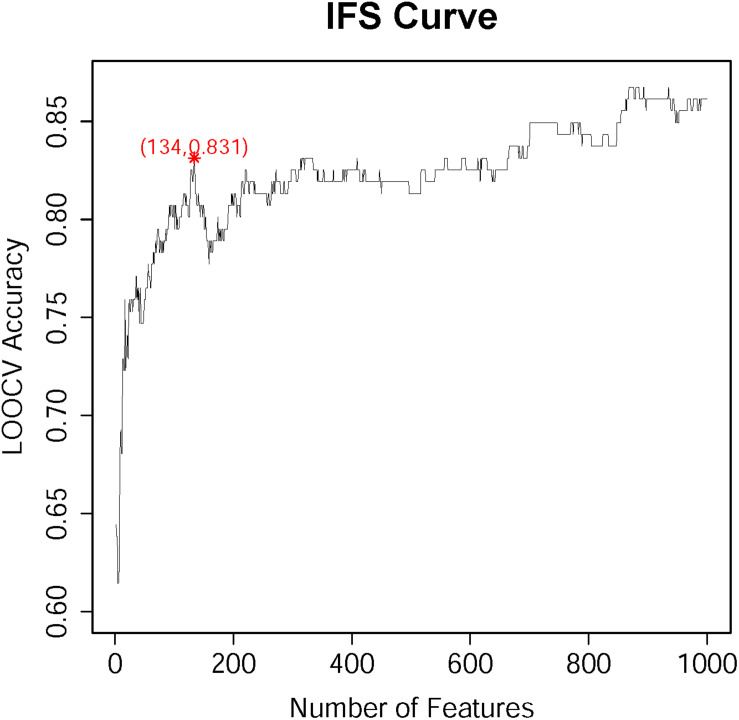
The IFS-curve obtained by IFS method. The *X*-axis represents the number of features participating in the classification. The *Y*-axis represents the LOOCV accuracy produced by SVM. The accuracy reached 0.831 when the top 134 features were used. When even more features were added, the accuracy did not increase too much. It reached the plateau area. Therefore, to balance the number of features and the accuracy, 134 features were selected.

According to the feature list obtained by the MCFS algorism, the IFS method was employed to identify optimal feature sets which could train the best performance for SVM. To save computing time, we established the series of feature subsets (*F*_1_, *F*_2_, *F*_3_, …, *F*_1000_) based on the top 1 to 1000 genes in *F*. For each feature set, we established a classifier by SVM algorithm and estimated optimal parameters through Leave-One-Out Cross-Validation (LOOCV). The LOOCV accuracies on multiple feature subsets were shown in Figure 1, from which we can see that the accuracy reached a plateau area when the top 134 features were used for building the classifier. The 134 optimal features were listed in Supplementary Table S2. The confusion matrix of the predicted results using the 134 features was shown in Table 1. It can be seen that all three classifiers had a great performance.

**TABLE 1:** The confusion matrix of the predicted results using the 134 features.

	Predicted D0*	Predicted D30**	Predicted Y1***
Actual D0*	47	12	6
Actual D30**	2	58	4
Actual Y1***	1	3	33

*, acute MI; **, 30-days post-MI; ***, 1-year post-MI.

### Cluster Analysis With Optimal Features

**FIGURE 2:**
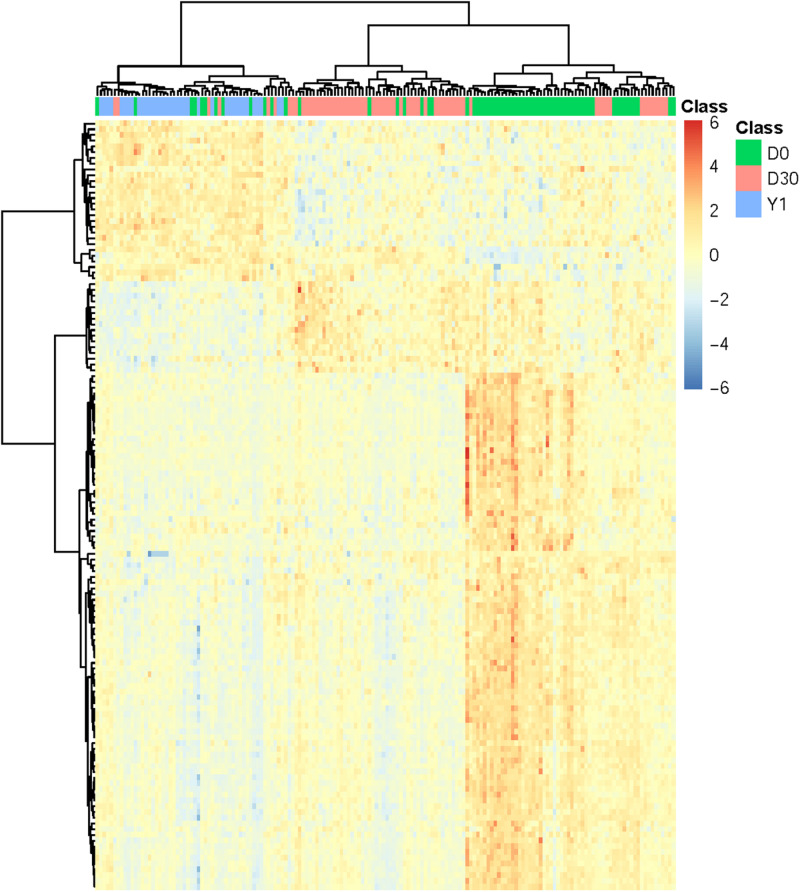
Heatmap of all MI samples on the top 134 genes. The columns refer to samples and the rows refer to genes. Different phases of samples were colored by green (D0 represents acute MI), red (D30 represents 30-days post-MI), and blue (Y1 represents 1-year post-MI), respectively. It can be seen that the samples from different time points had different expression patterns. For each time point, there was a corresponding cluster with highly expressed genes at this time point.

In order to confirm the performance of identified optimal features/genes representing different phases of samples, we performed cluster analysis on expression profiles of 134 optimal genes in 166 samples which incorporate three phases of MI (D0: acute MI, D30: 30-days post-MI, and Y1: 1-year post-MI). We used a heatmap to visualize the expression of such optimal genes among three groups of samples (Figure 2). The cluster tree illustrated that most samples belonging to the same phase can be clustered together and different phases were classified into different branches. In addition, these optimal genes were also classified into three clusters which correspond to high expression in three phases. The largest gene cluster with 90 genes was highly expressed in D0, the cluster with 16 genes had a high expression of D30, and the cluster with 28 genes was highly expressed in Y1.

**FIGURE 3:**
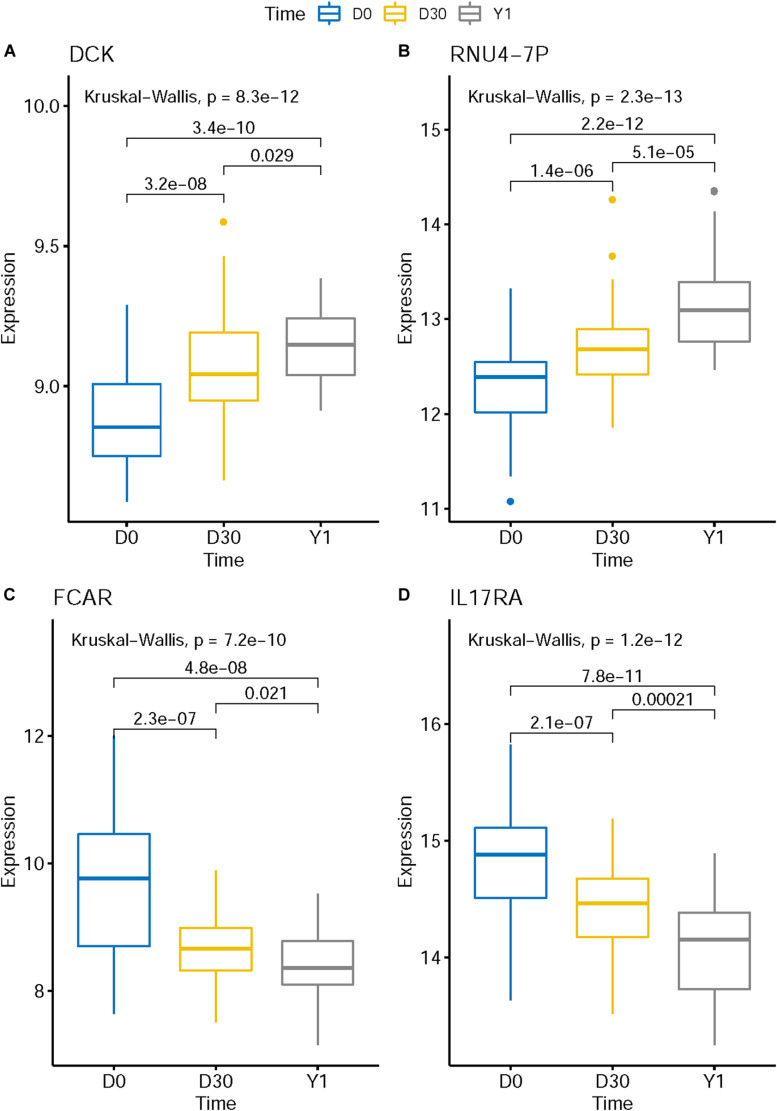
The boxplots of representative post-MI expression patterns. The expression level of genes like DCK **(A)** and RNU4-7P **(B)** increased in post-MI while the expression levels of genes like FCAR **(C)** and IL17RA **(D)** decreased in post-MI. These expression patterns may reveal the mechanisms of MI.

The expression levels of genes like KLHL8, HCLS1, MOB3A, IL17RA, ETF1, ZFAS1, CRK, MXD1, UBXN2B, FCAR, and EXTL3 decreased in post-MI while the expression levels of genes like DCK and RNU4-7P increased in post-MI. We plotted the boxplots of several representative genes in Figure 3. For example, in Figure 3C, the expression levels of FCAR on D0 was significantly higher than on D30 and the expression levels on D30 was significantly higher than on Y1. There was a consistent post-MI trend of FCAR. These expression patterns may reveal the mechanisms of MI. FCAR is a member of the immunoglobulin superfamily and encodes a receptor for the Fc region of IgA. The cell surface receptors for immunoglobulin, such as the protein of FCAR, can activate many inflammatory processes involved in atherosclerosis and coronary artery disease (Daëron, 1997; Gavasso et al., 2005). The variation in FCAR which causes an amino acid alteration was found to increase the risk of MI and coronary heart disease, indicating the potential functional role of FCAR in the development of cardiovascular disease (Iakoubova et al., 2006, 2008).

### Functional Enrichment Analysis on Optimal Features

We next performed functional enrichment analysis on these 134 optimal features/genes. A hypergeometric distribution test was applied to calculate *p* value to determine the significantly enriched entries. Firstly, we performed Gene Ontology enrichment analysis on the gene set. In biological progress aspect, the top 3 GO terms were GO: 0044264, GO: 0046903, and GO: 0005976, which correspond to cellular polysaccharide metabolic process, secretion, and polysaccharide metabolic process, respectively (Supplementary Table S3). The top GO term of cellular component was GO: 0005964, corresponding to phosphorylase kinase complex (Supplementary Table S4). The most significantly enriched GO term of molecular function was GO: 0004908, which was annotated to interleukin-1 receptor activity (Supplementary Table S5). Secondly, KEGG enrichment analysis was applied to discover the signaling pathways involved in these optimal genes. In this part, we found the insulin signaling pathway (hsa04910) was the top enriched KEGG pathway (Supplementary Table S6).

### Analysis of Gene Interaction Networks

**FIGURE 4:**
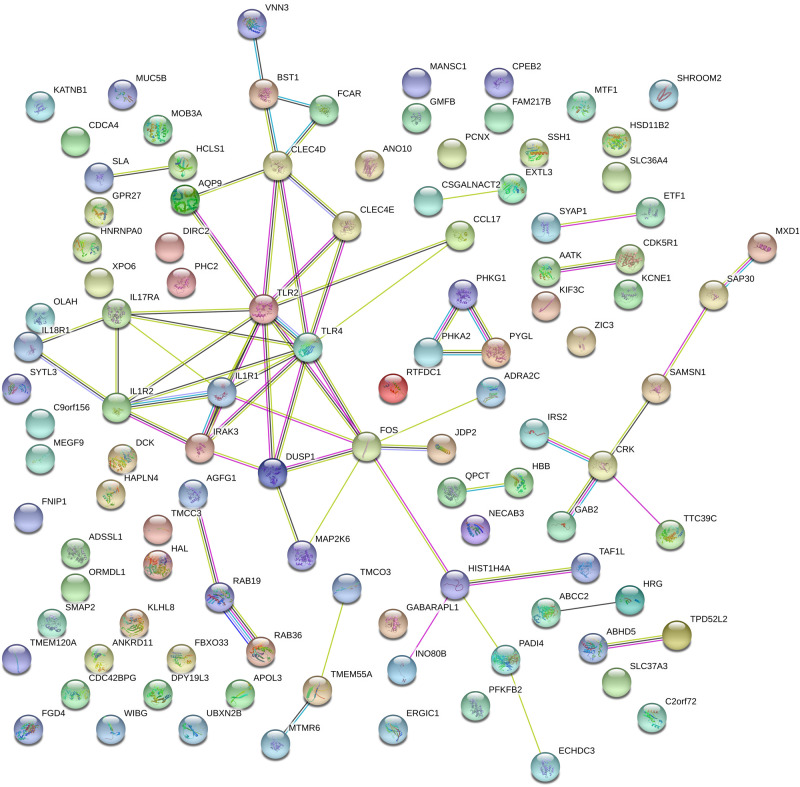
Gene networks containing IL1R1, TLR2, TLR4, and other related genes. These gene interactions were extracted from the protein-protein interaction network reported in a STRING database and plotted by the online drawing tool of STRING. IL1R1, TLR2, and TLR4 were located in the center positions and were hub genes.

To investigate the correlation of optimal genes, we applied gene interaction analysis on 134 features/genes to construct gene interaction networks. Proteins encoded by such classes of genes were input into a STRING database (Szklarczyk et al., 2018), mining interaction relationship. Although part of the genes showed no association with other genes, we found an interaction network consisting of dozens of genes and predicted three hub genes, including IL1R1, TLR2, and TLR4 (Figure 4), which may interact with each other to play a non-negligible role in the progression of MI.

IL1R1, TLR2, and TLR4 showed promising associations with MI. It was reported that the knockout of IL1R1 caused a reduction of leukocyte production after MI, leading to a decreased inflammation with better outcome (Sager et al., 2015). In another mice study, the up-regulated IL1R1 at 7 days post-MI prolonged the inflammation by suppressing neutrophil apoptosis (Iyer et al., 2015).

TLR2 plays a fundamental role in the activation of innate immunity (Binder et al., 2002). There are usually high levels of cytokines that result in inflammation in MI patients; TLR2 served as a key receptor to activate the corresponding pathways (Pagano et al., 2012). The experimental data indicated that circulatory TLR2 is relevant to different manifestations of myocardial I/R injury (Arslan et al., 2010). And the inhibition of TLR2 has beneficial effects on I/R injury in a murine model of MI (Arslan et al., 2009). TLR2 is the key receptor which can induce the inflammation after MI, therefore many MI-related genes show close interactions with TLR2.

TLR4 regulates the cytokines after cardiac damage (Arslan et al., 2010). Activation of TLR4 was related to myocytic inflammatory reaction in MI patients 14 days after onset, suggesting that TLR4 signaling plays a role in the progress after MI (Satoh et al., 2006).

## Discussion

### Optimal Genes Associated With Classification of MI

Using the feature selection, 134 genes were extracted and exhibited an excellent performance in our prediction model of SVM, suggesting that these genes may participate in the progression of MI. Here, we took some of the selected genes as examples to give a detailed discussion to validate the relevance of a given gene in distinguishing different pathological phases of MI. Through a literature review, several experimental evidences or analysis results have been found to confirm the reliability of our prediction.

#### DLGAP1-AS1

The top ranked feature identified by our computational analysis turned out to be DLGAP1-AS1, an RNA gene which is affiliated with the lncRNA class. A recent publication has reported that high expression of lncRNA DLGAP1-AS1 was detected in rats with acute ischemia-reperfusion (I/R) injury. And decreased DLGAP1-AS1 can alleviate vascular endothelial cell injury via PI3K pathway (Shen et al., 2020). The cause of I/R injury is mainly attributed to the reperfusion of the MI area, and vascular endothelial cells are the key defense with the occurrence of I/R injury (Carden and Granger, 2000; Causey et al., 2012). So, it came to the inference that down-regulated DLGAP1-AS1 serves as the protective regulator to mediate vascular endothelial cells in preventing I/R injury after the MI. This builds relevance for the alteration in DLGAP1-AS1 expression in the progression of MI. Besides that, gene DLGAP1 showed significant differential expression in Flk-1 knockout mice under the treatment of heart perfusion (Thirunavukkarasu et al., 2008). Flk-1 is one of the most important receptors that trigger cardioprotective signals and plays a crucial role in I/R injury (Shalaby et al., 1995; Addya et al., 2005), as DLGAP1-AS1 can target DLGAP1 and regulate its expression. This finding provided further support to suggest DLGAP1-AS1 was closely related to the progression of MI.

#### PYGL

The following ranked gene was Glycogen Phosphorylase L (PYGL), which encodes a homodimeric protein that is involved in galactose metabolism (Tomihira et al., 2004). Early research has mentioned the application of glycogen phosphorylase in the diagnosis of myocardial ischemic injury and infarction (Krause et al., 1996; Mair, 1998). Recently, PYGL was reported to display an up-regulated expression in an acute MI cohort compared to normal controls (Zhang et al., 2017). Another study has demonstrated that up-regulated PYGL may induce the RIP1-dependent necrosis after I/R injury, implying that PYGL is associated with the subsequent progress after AMI and I/R injury (Oerlemans et al., 2012). This evidence proves our prediction results were reasonable.

#### MEGF9

MEGF9 was also identified as an important gene related to the classification of MI. MEGF9 is a protein coding gene and is associated with Fiedler’s Myocarditis disease. Some studies have observed differentially expressed MEGF9 and identified it as the key gene involved in AMI and MI (Cheng et al., 2017; Qiu and Liu, 2019). As demonstrated by genome-wide linkage analysis in autosomal dominant congenital heart defects, the risk region in chromosome 9q was found and MEGF9 turned out to be one of the candidate genes in this position. However, no mutations were found in this gene through the sequence analysis, suggesting that MEGF9 may play its role by post-transcriptional regulation instead of at the genome level (Van De Meerakker et al., 2011). Hence, the specific expression pattern could be a signature for diagnosing MI and even distinguishing different phases of MI.

#### PHC2

Next, another gene called PHC2, which is associated with the metabolism of proteins, was selected by our computational analysis. PHC2 was reported as one of the differentially expressed genes in patients with MI compared to controls by bioinformatics screening (Wu et al., 2018). Another study also confirmed the key role of PHC2 in the pathogenesis of MI through protein-protein interaction network analysis (Qiu and Liu, 2019). These results implied that PHC2 may act as a hub gene which can mediate some other genes’ interaction and regulate downstream pathways, and then influence the progress of MI. Our analysis highlighted the importance of PHC2, pointing out that this specific gene may be applied as a marker for the prediction of recurrent MI.

Through literature review and reasonable inference, the selected genes mentioned above were all found to play crucial roles in the progress of MI and show the discriminative ability to indicate the pathological degree of disease. It validated the reliability of our prediction model. Considering the length limitation of the article, we can’t give extended descriptions of all 134 selected genes. We believed that these 134 selected genes were meaningful during the development of MI and its subsequent progression, and they will contribute to the research of molecular mechanism and provide benefits for the therapy of disease.

### Gene Ontology Enrichment Analysis

Given that the selected 134 genes were deemed as important features for the classification of different phases of MI, we performed GO and KEGG functional enrichment analysis to explore the key biological processes or pathways during the progress of disease. As shown in Supplementary Tables S3–S6, we analyzed the enriched GO terms and KEGG pathways which showed statistical significance. A detailed discussion was given about the linkage between certain functional sets and MI.

Based on the enrichment results of 134 selected genes, we found some GO biological process terms with high scores turned out to be involved in the polysaccharide metabolic process, including GO: 0044264 and GO: 0005976. As early as 1965, scientists have noticed the important role of glucose load in MI (Cohen and Shafrir, 1965). Recent studies reported that certain polysaccharide compounds can affect myocardial injury via regulating the inflammation response (Li et al., 2011; Lim et al., 2016). As demonstrated by experiments on rat, the polysaccharide extract from Momordica charantia down-regulated the expression of NF-kappaB and ameliorated oxidative stress and inflammation, which caused a cardioprotective effect against MI (Raish, 2017). Polysaccharide metabolism plays an important role during the progression of MI, so the biological processes related to polysaccharide metabolism are meaningful and can be used to indicate the progression of disease based on its specific pattern.

Apart from GO terms that belong to biological processes, we found these 134 genes are also enriched in a cellular components term GO: 0005964 with the highest probability. GO: 0005964 refers to phosphorylase kinase complex. For cardiomyocytes, the storage of glycogen is important during the emergency situation. Increasing Ca^2+^ concentration in cytosol can induce glycogenolysis by the activation of phosphorylase kinase, which can alleviate myocardial damage during MI or cardiac surgery (Raish, 2017). In fact, some phosphorylases have been applied in the diagnosis of myocardial ischemic injury and infarction since the serum level of phosphorylase showed a signature with the diseases (Krause et al., 1996). It is reasonable for the MI-related genes to be enriched in such GO term that would mean the phosphorylase play a crucial role during the progression of MI.

The most enriched GO terms of molecular function turned out to be interleukin-1 (IL-1)-related functions including GO: 0004908 and GO: 0019966, which represent IL-1 receptor activity and IL-1 binding, respectively. An interleukin-1 receptor gene ST2 was increased in the serum after MI, suggesting that this gene may participate in innate immunity during myocardial injury (Weinberg et al., 2002). What’s more, ST2 was reported to be able to predict the clinical outcome in AMI due to its role in cardiac pathophysiology (Shimpo et al., 2004). Many publications have observed the elevated serum level of IL-1 receptor in patients with AMI (Shibata et al., 1997; Balbay et al., 2001). These findings proved the important role of IL-1 in the progression of MI, and confirmed the relation between selected genes and MI.

### KEGG Pathways Enrichment Analysis

The KEGG pathways enrichment analysis provided various pathway results. Among these, the highest enriched pathway turned out to be hsa04910, which is an insulin signaling pathway. Increased insulin can promote the metabolism of glucose to maintain the balance of blood glucose. The connection between abnormal insulin signaling and heart disease has already been reported, in that diabetes mellitus significantly increased the risk of ischemic heart disease (Miettinen et al., 1998). Insulin can protect cardiomyocytes from apoptosis through activating downstream pathways such as PI3K and Akt (Yao et al., 2014). It was reported that impaired insulin signaling will cause the dysfunction of mitochondria after MI due to the reduced glucose transport and oxygen content (Sena et al., 2009). Thus, the insulin signaling pathway is important during the progression of MI and influences the pathological degree of disease.

## Conclusion

Taken together, the gene features yielded by our model showed strong relevance to the pathological progression of MI, suggesting their discriminative ability in the classification of different phases of disease. This validated the reliability of our machine learning model and proved that it can be used as a novel approach to predict the status of MI patients. Our work will contribute to the precise diagnosis and help to decide on the optimal treatment for each patient with MI. In addition, the genes identified by our analysis provided new understanding about the pathogenesis of MI and established a solid foundation for future research.

## Data Availability Statement

The datasets generated for this study can be found in the GSE123342.

## Author Contributions

HH and QL contributed to the conception and design. HH, ML, and FC contributed to the development of methodology. All authors contributed to analysis and interpretation of data, writing, review, and/or revision of the manuscript.

## Conflict of Interest

The authors declare that the research was conducted in the absence of any commercial or financial relationships that could be construed as a potential conflict of interest.

## Footnotes

1 http://www.ipipan.eu/staff/m.draminski/mcfs.html

2 https://cran.r-project.org/web/packages/e1071/index.html

## Supplementary Material

TABLE S1 The ranked features with RI values.

TABLE S2 The 134 optimal features for building the classifier.

TABLE S3 Gene ontology enrichment analysis for biological progress aspect.

TABLE S4 Gene ontology enrichment analysis for cellular component.

TABLE S5 Gene ontology enrichment analysis for molecular function.

TABLE S6 KEGG enrichment analysis for discovering the signaling pathways involved in these optimal genes.
